# Fibrinogen post‐translational modifications are biochemical determinants of fibrin clot properties and interactions

**DOI:** 10.1111/febs.17236

**Published:** 2024-08-23

**Authors:** Margarita Tenopoulou

**Affiliations:** ^1^ Laboratory of Biochemistry, Department of Chemistry University of Ioannina Greece

**Keywords:** fibrin clot properties, fibrinogen, post‐translational modifications, thrombosis

## Abstract

The structure of fibrinogen and resulting fibrin formed during the coagulation process have important biological functions in human physiology and pathology. Fibrinogen post‐translational modifications (PTMs) increase the complexity of the protein structure and many studies have emphasized the potential associations of post‐translationally altered fibrinogen with the formation of a fibrin clot with a prothrombotic phenotype. However, the mechanisms by which PTMs exert their action on fibrinogen, and their causal association with disease pathogenesis are relatively unexplored. Moreover, the significance of fibrinogen PTMs in health has yet to be appreciated. In this review, the impact of fibrinogen PTMs on fibrinogen functionality is discussed from a biochemical perspective, emphasizing the potential mechanisms by which PTMs mediate the acquisition of altered fibrinogen properties. A brief discussion on dysfibrinogenemias of genetic origin, attributed to single point variations of the fibrinogen molecule is also provided, highlighting the influence that amino acid properties have on fibrinogen structure, properties, and molecular interactions that arise during thrombus formation.

## Graphical abstract

The biochemical and biophysical properties of fibrinogen and fibrin, which is formed during coagulation, are critical for proper hemostatic function. The formation of abnormal fibrin clots has been linked to a prothrombotic phenotype and disease states. Here, the impact of post‐translational modifications (PTMs) upon fibrinogen functionality is discussed with an emphasis on the potential mechanisms by which PTMs mediate the acquisition of altered fibrinogen and fibrin properties.

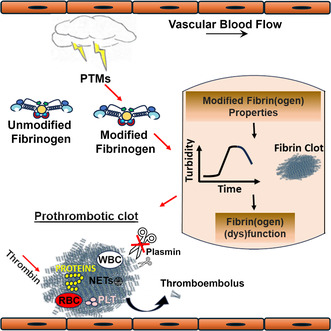

**Abbreviations**
CAD: coronary artery disease
HTL: homocysteine thiolactone
MCO: metal‐catalyzed oxidation
PTM: post‐translational modification
RNS: reactive nitrogen species
ROS: reactive oxygen species
VTE: venous thromboembolism

## Introduction

Circulating fibrinogen is a critical component of the blood coagulation system. During the last step of the coagulation cascade, thrombin‐activated soluble fibrinogen is irreversibly converted to insoluble fibrin polymer, the structural component of the blood clot that was first described in 1666 by Malpighi [1]. Fibrinogen acts as a provisional matrix for recruiting leucocytes and as a reservoir for growth factors and participates in wound healing by promoting cell migration, re‐epithelialization and angiogenesis, as well as in inflammation and host defense process against microbes [2, 3, 4, 5, 6] and ref therein. Fibrin removal is an essential process for the progression of the wound healing and tissue regeneration procedure. Formation and breakdown of fibrin clots involve precise and concerted interactions between coagulation and fibrinolytic factors and any disruption leads to obstruction of blood flow associated with thromboembolic events or bleeding episodes.

**Fig. 1:**
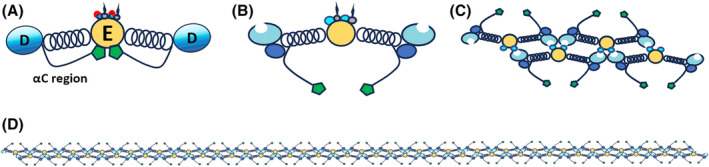
A diagrammatic representation of fibrinogen and fibrin polymeric molecules. (A) Fibrinogen basic structure: Central domain (E region), Fibrinopeptides A (red cap), Fibrinopeptides B (black cap) in E region, distal globular domain (D region), C‐terminal part of Aα‐chain (αC region). (B–D) Initial fibrin structures formed upon thrombin cleavage: (B) fibrin monomer, (C) double‐stranded fibrin oligomers, (D) protofibrils (20–25 monomers in length).

Human fibrinogen is a 340 kDa protein primarily synthesized in the liver and secreted into circulation. The typical range of fibrinogen concentration in the blood is 2–4 g·L^−1^. For a detailed fibrinogen structural information, a comprehensive review is provided by Litvinov *et al*. [7]. Briefly, structurally the molecule consists of two identical subunits each of them is composed of three non‐identical polypeptide chains namely Aα (610 amino acids), Bβ (461 amino acids), and γ (411 amino acids) interconnected with 29 disulfide bonds. All three chains are interconnected with their amino‐terminals (NH_2_‐terminal) at a central domain termed E region (Fig. 1). The carboxy‐terminal (C‐terminal) parts of the Ββ‐ and γ‐chains form the β‐ and γ‐module, respectively, both comprising a globular domain referred as D region [8, 9]. E region is connected to each D region through a triple coiled‐coil segment with most of the non‐polar amino acid side chains directed inward [10, 11]. Both E and D regions contain essential sites indispensable for fibrin clot formation. The C‐terminal part of Aa chain (aC region) contains the aC domain which is tethered to fibrinogen molecule through an extending flexible aC‐connector [12, 13]. When fibrinogen is in its inactivated form the aC domains interact intramolecularly with each other and with the central region of the molecule, associations that are disrupted upon activation by thrombin action allowing αC–αC interactions between different fibrinogen entities [12]. αC domains are highly polar and hydrophilic [14, 15] whereas aC‐connector consists of polar and non‐polar amino acid residues [11].

**Fig. 2:**
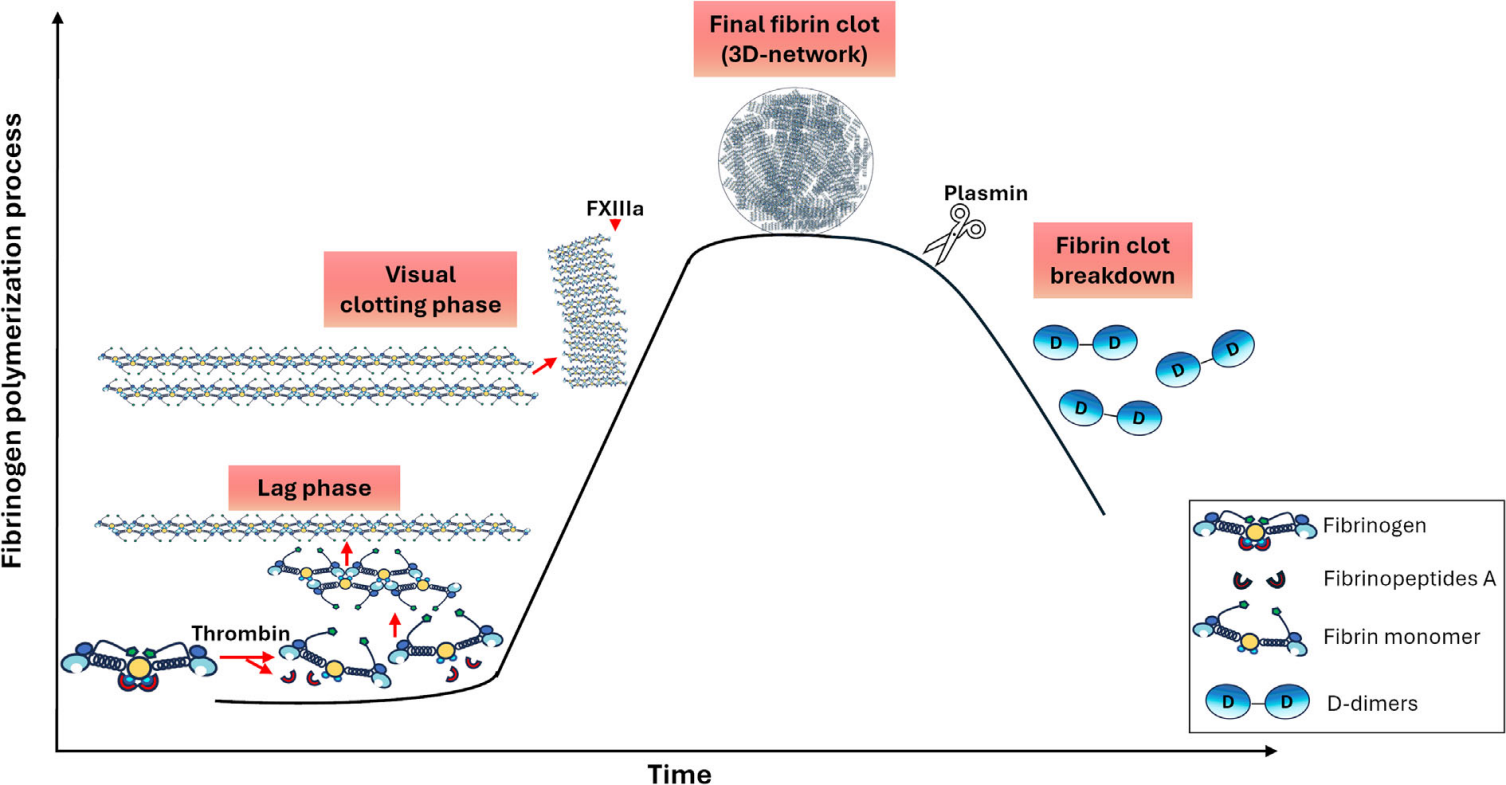
Schematic representation of the critical stages during fibrinogen polymerization, fibrin clot formation and lysis. During the lag phase, fibrinopeptides A are released from fibrinogen by the action of thrombin to generate fibrin monomers which in turn arrange into polymeric molecules until they reach the protofibril length of 20–25 monomers. In the visual clotting phase, protofibrils undergo rapid lateral aggregation, fibrin polymers are stabilized by factor FXIIIa and fiber bundles are formed, culminating in a 3D network of branching fibers. Fibrinolysis follows and blood clot dissolves by the action of plasmin which cleaves fibrin at specific sites generating fibrin degradation products such as D‐dimers.

Below, the molecular structure and function of fibrinogen and fibrin polymeric molecules is briefly described (Fig. 1) as well as the essential steps of its conversion to fibrin polymer (Fig. 2). The readers are referred to excellent and comprehensive reviews regarding these topics [7, 8]. We also discuss the development of abnormal fibrin clot due to genetic variations of fibrinogen. Existing literature is comprehensively presented and an extensive discussion is provided addressing the idea that PTMs represent biochemical determinants of fibrin clot properties as well as molecular and cellular interactions.

## Fibrin clot formation process

Fibrin polymerization is a multi‐step process which proceeds through a series of consecutive steps as it is shown in Fig. 2 and described below.

### Fibrinogen cleavage

Initially, thrombin cleaves fibrinopeptide A from the soluble fibrinogen NH_2_‐terminal of Aα‐ chain exposing a positively charged sequence (also known as knob A) with the sequence motif Gly‐Pro‐Arg (GPR). This process is essential for the generation of fibrin monomers. Monomers are 45 nm long structures [7].

### Fibrin polymerization

Newly exposed GPR sequence strongly and stably binds by electrostatic interactions to a negatively charged complementary site (hole a) located within the γ module of the γ‐chain of an opposing fibrin monomer [16]. Two monomers join by end‐to‐end alignment (by their D regions of the γ modules) along with a third molecule via D:E interactions and drive the formation of a double‐stranded element which continue to grow longitudinally until it reaches a length of a double‐stranded protofibril [7, 17, 18, 19]. At this stage, fibrinopeptide B is enzymatically released from the NH_2_‐terminal of Bβ‐chain exposing the sequence motif Gly‐His‐Arg (GHR) (knob B) which, similarly to knob A, binds to a complementary site (hole b) within the β module of the Bβ‐chain of another monomer. Although not essential for the formation of fibrin clots, unmasking of knob Β polymerization site results in a more rapid fibrin assembly [18]. Cross‐linking of γ‐chains and a‐polymers formation are simultaneously taking place by the action of thrombin‐activated FXIII conferring stability to the molecule [20].

### Fibrin assembly

Spontaneous lateral association of protofibrils into fiber strands increases fiber diameter [8]. Subsequent fibrin branching results in a 3‐dimensional space‐filling network [21, 22].

Although the mechanism of protofibril aggregation is less clear, there is evidence suggesting that aC regions [23, 24] and knob Β:hole b interactions are considerable players in the process by enhancing fibrin assembly during polymerization. Low‐affinity calcium‐binding sites formed by residues of the β‐ and γ‐chains may also be involved in this process [25]. During fibrin clot formation, fibrinogen acquires specific properties influenced mainly by its milieu. The contribution of fibrinogen to the biological processes in which it is implicated largely depends not only on its properties, but also on interactions between specific‐binding sites on fibrinogen and proteins including zymogen plasminogen and its activator (known as tissue plasminogen activator, tPA) as well as factor FXIIIa. FXIIa mediates fibrin cross‐linking providing stability to the clot and also facilitates the incorporation of antifibrinolytic proteins (plasminogen activator inhibitor 2, α2‐antiplasmin, thrombin‐activable fibrinolysis inhibitor) into the fibrin network contributing to its resistance to fibrinolysis [26, 27, 28]. Additional fibrin(ogen) (collectively for fibrinogen and fibrin) interactive sites involve association with non‐coagulation proteins such as fibronectin, growth factors and others [a list of fibrin(ogen)‐binding plasma proteins along with indications of the biological significance are provided by Weisel and Litvinov] [8]. Fibrin network also interacts with cells including neutrophils, monocytes, leukocytes, fibroblasts, platelets (PLT), and endothelial cells lining the vessel walls by direct binding through cell surface receptors. Fibrin has also been colocalized with neutrophil extracellular traps (NETs) [23, 29].

**Fig. 3:**
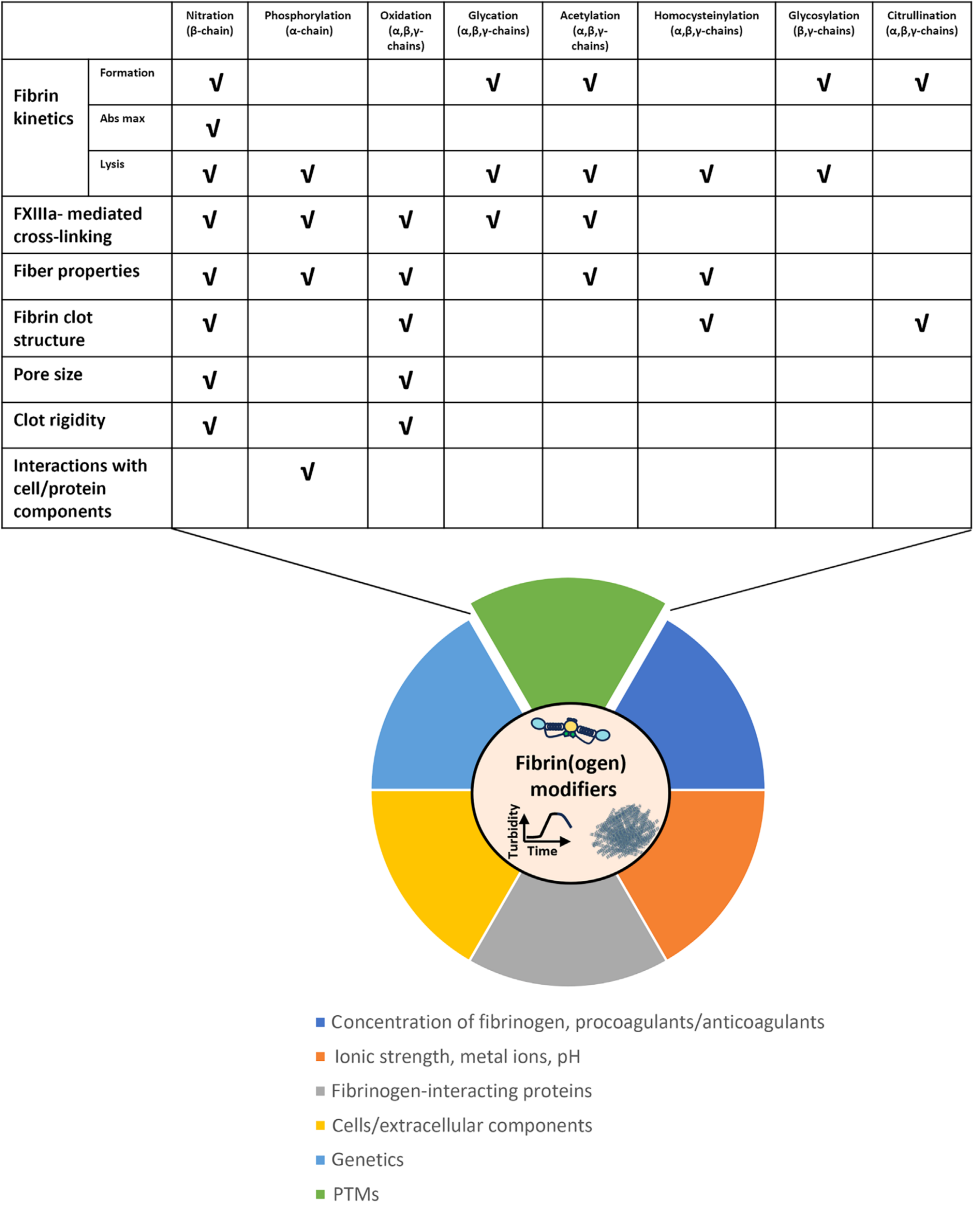
Key factors that modulate fibrin clot formation, structure, and determine clot properties. Local concentration of fibrinogen and procoagulants/anticoagulants, metal ions, pH, ionic strength, fibrinogen‐interacting proteins, cells/extracellular components, genetics, PTMs are among the critical factors affecting fibrin(ogen) properties (shown by different colors in pie‐chart). Table contains information regarding PTMs and the fibrinogen chains that accommodate those PTMs along with functionality effects.

After completion of its biological function, fibrin is removed in a systematic way by the fibrinolytic system which involves plasminogen activation by tPA and eventually activation of plasmin protease. Plasmin cleaves fibrin on specific lysine residues creating additional binding sites for tPA and plasminogen reinforcing the fibrinolytic process [8, 30, 31]. Fibrinolysis results in the formation of cross‐linked fibrin degradation products with the smallest being the D‐dimer formed of two D regions of two adjacently connected fibrin monomers (Fig. 2). An imbalance in the aforementioned functions and interactions may influence the formation of the final fibrin network and its acquired properties promoting the development of a clot with a prothrombotic phenotype. A prothrombotic clot phenotype presents with unfavorably altered fibrin clot properties, characterized by accelerated formation of dense fibrin clots, highly branched networks with thin fibers and small size of pores in fibrin networks that display impaired fibrinolytic degradation and associates with pathological states such as thrombosis and thromboembolism [32, 33]. Fibrin properties include biochemical (kinetics of fibrin polymerization and lysis) and structural properties (structure of individual fibers, architecture of the fibrin matrix, clot porosity) whereas as a viscoelastic biopolymer [34] fibrin expresses mechanical properties allowing to deform appropriately under deformation‐inducing states. Numerous studies have focused on factors that govern fibrinogen conformation, fibrin formation, structure, and stability whereas abnormally formed clots are associated with pathology ranging from CAD and thromboembolism to chronic kidney disease and cirrhosis [9, 35]. These factors are summarized in Fig. 3.

## The impact of hereditary dysfibrinogenemia on fibrinogen properties

Experimental evidence links inherited variants of fibrinogen with thrombotic or bleeding phenotype in affected individuals documenting that a genetic component contributes to fibrinogen characteristics. Systematic and detailed descriptions of heritable fibrinogen variants can be found at Online Mendelian Inheritance in Man (omim.org) and Groupe d'etude sur l'hemostase et la thrombose (geht.org).

Some notable variants include substitution of arginine. Substitution of arginine at position 16 in the fibrinopeptide A cleavage site with cysteine (AαR16C), or histidine (AaR16His) resulted in reduced polymerization rate, due impaired release of fibrinopeptide A [36, 37, 38]. Affected patients with either substitution were clinically presented with significant hemorrhagic diathesis or thrombotic phenotype.

Fibrinogen Paris V (alternative names Chapel Hill III or Dusart fibrinogen) describes a mutation located in a hydrophilic sequence in fibrinogen Aa chain which is involved in lateral aggregation of protofibrils. More specifically, substitution of arginine 554 to cysteine (AaA554C) is characterized by functional anomaly of circulating fibrinogen, associated with abnormal fibrin polymerization, formation of thinner than normal fibers and tightly packed, and clots with decreased porosity. Thrombotic episodes in affected individuals most likely are caused by impaired clot thrombolysis due to the abnormal clot structure [39, 40, 41, 42]. Collet *et al*. [40], proposed that this amino acid substitution induces conformational changes of the aC domain leading to a less flexible fibrinogen molecule as well as impacts the intermolecular interactions mediated through aC domains.

Fibrinogen Naples (or MILANO II) is characterized by a homozygous substitution of alanine, a small size and hydrophobic amino acid at position 68 in Bβ‐chain with threonine (BβΑ68T), a medium size and polar amino acid. The phenotypic result of this mutation is defective thrombin‐binding and thrombophilia [43].

Structural analysis of another genetic variant in the Bβ‐chain revealed that an arginine residue at position 166 plays a crucial role in fibrin lateral aggregation [44]. In dysfibrinogenemia Longmont, presented with arginine substitution by cysteine (BβR166C) [45] fibrinogen molecule was found disulfide‐bridged with either the homologous cysteine of another mutated fibrinogen molecule or with protein‐free cysteine. However, the dimeric forms of abnormal fibrinogen are unlikely to contribute to the reported polymerization defect since dimer removal did not restore the dysfunction. Disulfide bridges of Cys166 with free cysteine may represent a mechanism which negatively impacts interaction of fibrinogen Longmont during coagulation and thus predisposes to hemorrhagic events.

Abnormal fibrin clot structure and delayed fibrinolysis were observed in fibrinogen variant BβP235L. Morris *et al*. [46], reported conformational changes in this fibrinogen variant and the defect has been associated with chronic thromboembolic pulmonary hypertension in affected patients.

In cases of inherited dysfibrinogenemia due to variants of γ‐chain, DNA sequencing revealed substitutions of the arginine 275 to the medium size and polar amino acids histidine (Barcelona III and IV), cysteine (Villajoyosa) or serine which results in alterations of fibrinogen function, with difference in severity though [47]. This arginine residue is indispensable for normal D:D interactions [48]. Borrell *et al*., demonstrated altered properties in fibrin monomers produced from fibrinogen isolated from Arg275His or Arg275Cys individuals. Nevertheless, all three variants were characterized by delayed fibrin polymerization. Low turbidity of fibrin clots but unaffected interaction with t‐PA or plasminogen were observed in the first two variants. These results suggest that arginine 275 is basically important in maintaining the protein structure essential for normal polymerization.

In fibrinogen Ale's, the amino acid exchange of aspartate residue at position 330 in γ‐chain, a medium size and acidic amino acid to a medium size and hydrophobic amino acid valine (Asp330Val) impairs fibrin polymerization by compromising the interaction of complementary Knob A:hole a sites [49]. A similar impairment was also observed in fibrinogen Baltimore in which γ292Gly is substituted with the medium size and hydrophobic amino acid valine [50] which was associated with defective fibrin polymerization most likely attributed to the lost integrity of the particular γ‐chain region involved in fibrin assembly.

The fibrinogen Thr312Ala single‐nucleotide polymorphism in aC domain is located in an area that FXIIIa interacts with fibrinogen and leads to altered FXIIIa cross‐linking and the formation of stiffer clots with thicker fibrin fibers associated with venous thromboembolism (VTE) [51, 52].

Using recombinant fibrinogen with a point substitution of lysine 405 to arginine located in the FXIIIa cross‐linking sites on γ‐chain responsible for γ‐dimer formation, Standeven *et al*. [53] were able to show the significant influence of the amino acid substitution to fibrin viscoelastic properties.

The data above strongly suggest that amino acid substitutions with neo‐amino acids bearing new biochemical properties are important mediators of protein structure and functionality.

## Fibrinogen PTMs as biochemical influencers of fibrinogen acquired properties

Studying the congenital dysfibrinogenemia allows to understand better the relationship of amino acid biochemistry and protein structure and function. It is not uncommon that the function of a protein is, at least in part, impacted by covalent modifications on selected amino acid residues named post‐translational modifications (PTMs). PTMs have the potential to alter stereochemical and/or electrostatic properties of a protein and often have important regulatory functions ensuing protein‐to‐protein interaction, determine protein stability and localization as well as finely tuning enzymatic activity [54, 55]. However, PTMs may influence protein negatively promoting pathological states such as neurodegenerative and cardiovascular diseases (CVD). Several studies document that fibrinogen is targeted by PTMs mostly through oxidative chemistries and these modifications are functionally associated with an acquired type of dysfibrinogenemia. It has to be noted that the majority of existing studies investigate the impact of a single PTM and relates it with conformational changes as well as with changes on fibrinogen kinetics during clotting and clot breakdown, and altered fibrin clot characteristics.

**Fig. 4:**
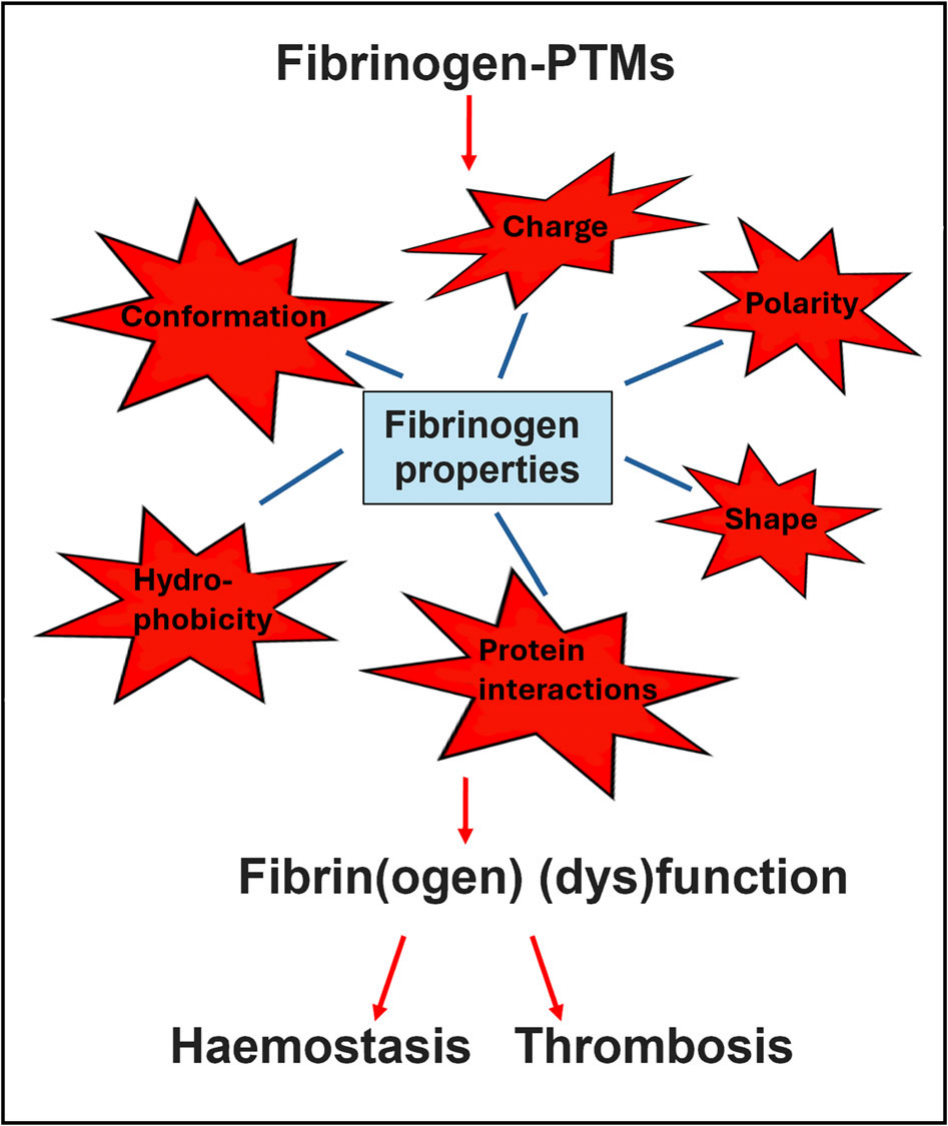
Possible biochemical alterations of fibrinogen molecule mediated by PTMs. PTMs may have an impact on fibrinogen by affecting its charge, polarity, hydrophobicity and molecular flexibility, and therefore its shape, conformation and protein interactions.

The current review focuses on the premise that PTMs alter fibrinogen structure, function, and properties. It provides a comprehensive coverage of existing knowledge but also introduces and discusses novel ideas regarding this premise. The potential biochemical alterations of fibrinogen molecule induced by PTMs are illustrated in Fig. 4.

## Experimentally‐identified fibrinogen PTMs

### Oxidation

Reactive oxygen species (ROS) are implicated in pathophysiological processes such as aging, neurogenerative diseases, CVD including thrombosis, and other. ROS are also known modifiers of amino acid side chains and oxidative PTMs alter protein structure and function, propagate or inhibit signal transduction. Among plasma proteins, fibrinogen is a major target to oxidative modifications [56]. Functional consequences of fibrinogen due to oxidation lead to loss‐of‐function. Oxidative modifications of fibrinogen and effects on fibrin structure and function have been previously reviewed by Martinez *et al*. [57]. Metal‐catalyzed oxidation (MCO) occurring only in α‐ and β‐chains resulted in the formation of dityrosine as well as the carbonylation of histidine, proline, arginine, and lysine residues [58, 59, 60]. It has been shown that oxidative modifications induce conformational changes which negatively impact the binding of fibrinogen to platelet receptor causing reduced platelet adherence and aggregation [61]. However, the latter has been debated [62]. In addition, it has been shown that MCO led to inhibition of fibrinogen clotting due to impaired fibrin monomer polymerization [56]. Rosenfeld *et al*. [63] reported that ozone‐oxidized fibrinogen demonstrated accelerated FXIIIa‐mediated cross‐linking of γ and α polypeptide chains compared to the original fibrinogen and enhanced D:D interaction. Fibrinogen oxidation by hypochlorite, HOCl, (a physiologically generated oxidant in the body especially under inflammatory conditions) results in a dose‐dependent loss‐of function as is evidenced by the reduced kinetics of fibrin polymerization reflecting the inhibition of lateral association of protofibrils. Oxidizing conditions generated through myeloperoxidase and hydrogen peroxide impaired fibrin polymerization, consistent with the aforementioned exposure to HOCl. Hypochlorite exposure also resulted in the oxidation of a wide variety of amino acids including tyrosine, lysine, proline, aspartate, asparagine, tryptophan, histidine, phenylalanine, serine, glutamate, and arginine located in all three fibrinogen chains. Notably, the modified amino acids do not locate regions responsible for fibrinogen conversion to fibrin, however, their oxidation is still able to hinder lateral aggregation of protofibrils with a still unknown mechanism [64, 65]. The sulfur‐containing amino acid, methionine, is readily oxidized by ROS to form methionine sulfoxide due to the low oxidative potential of sulfur [66]. This modification, a two electrons oxidation process, is induced by oxidants generated in biological systems such as peroxynitrite, hydrogen peroxide, hypochlorous acid [67] and is associated with alteration of protein structure and functional consequences [68]. Hypochlorous acid was shown to preferentially oxidize γ‐Met78, Bβ‐Met367, and Aα‐Met476 residues on all three chains of fibrinogen and was associated with the formation of a dense structure of thin fibers with decreased stiffness and viscosity and delayed fibrinolysis most probably due to structural modifications [69]. In particular, methionine 476 is located within the αC region of the Aα‐chain which contributes to lateral aggregation of fibrin fibers and thus this modification may contribute to altered lateral aggregation. In this regard, Bychkova *et al*. [70], reported that the αC region was the most prone site to ozone‐induced oxidation than the rest structural elements in Aα‐chain which might be responsible for inhibiting lateral aggregation of protofibrils. Since methionine oxidation is enzymatically reversed *in vivo* [71], oxidation/reduction of fibrinogen may represent a mechanism of controlling hemostasis in biological systems. The above‐mentioned data implicate fibrinogen oxidative modifications in the thrombotic process.

Protein carbonylation, the formation of carbonyl groups (C=O) on side chains of some amino acids such as lysine, arginine, is another post‐translational modification related to oxidative stress [72]. Recent work from Błaż et al. [73] linked elevated plasma protein carbonyl levels in patients with acute ischemic stroke with prothrombotic fibrin clot properties and associated them with stroke severity. Moreover, results from other studies showed that fibrinogen is susceptible to carbonylation, and increased fibrinogen carbonyl levels have been measured in subjects of chronic inflammation such as smokers, patients suffering from inflammatory diseases as well as patients with subacute phase of myocardial infarction [74, 75, 76]. These results provide evidence for the impact of carbonylation as a mechanism of fibrinogen (dys)function and altered fibrin properties that may associate with unfavorable outcomes in patients with CAD [77, 78].

### Phosphorylation

Protein phosphorylation, induced by the enzymatic transfer of a phosphate group to the hydroxyl group of serine, threonine, or tyrosine, represents a global signaling mechanism in cells. Witt’s group identified two endogenous phosphorylation sites at AaSer3 and AaSer345 residues using purified human fibrinogen. Although the functional impact of phosphorylation was unknown the authors speculated based on location, that phosphoserine 345 either facilitates a specific function of the extremely polar zone in Aα‐chain, or it may interfere with FIIIXa binding and Aα‐chain cross‐linking. The levels of phosphorylation of human fibrinogen were found to increase under acute phase conditions resembling the range of phosphorylation reported for fetal fibrinogen followed by a decline suggesting a dynamic regulation of fibrinogen phosphorylation [79]. Additional studies performed during the same decade revealed potential sites of phosphorylation in central and C‐terminal part of Aα‐chain and/or γ‐chain in regions that may affect fiber thickness, fibrinolysis sensitivity, and molecular and cellular interactions [80, 81]. Interestingly, *in vitro* experiments showed that the phosphorylation‐dependent fiber thickness was also affected by dephosphorylation. On the contrary, phosphorylated fibrinogen isolated from patients admitted for hip‐replacement surgery displayed decreased susceptibility to plasmin degradation which was not influenced by the removal of phosphate content [82, 83, 84]. These results prompted Martin and Björk to propose that phosphorylation induces conformational changes enhancing the extend of ordered secondary structure. Nonetheless, the kinases and phosphatases that mediate fibrinogen phosphorylation and dephosphorylation respectively still remain unknown [83].

### Tyrosine nitration

Of the well‐documented fibrinogen PTMs from a biochemical standpoint and with potential biological significance is tyrosine nitration, the selective covalent addition of a nitro group (‐NO_2_) on carbon at position 3 of the phenolic ring of tyrosine residues forming 3‐nitrotyrosine (3‐NT). Nitrated proteins have been found in animal and cellular models of disease and in various human diseases including inflammatory, neurodegenerative, cardiovascular disorders, ischemic stroke and metabolic disorders [85, 86, 87, 88]. Protein‐bound 3‐nitrotyrosine represents an antigenic neoepitope recognized by specific immunoglobulins in coronary artery disease (CAD) [89]. Tyrosine nitrated proteins are enriched in atheromatous lesions and in circulation of CAD subjects. Elevated levels of plasma nitrated fibrinogen have been documented in CAD patients as compared to healthy controls and in patients with VTE as compared to non‐VTE subjects [90, 91]. Relevant chemistry and biological pathways regarding the *in vivo* formation of 3‐NT have been described in previous comprehensive reviews [86, 92]. Several potential *in vivo* nitrating agents have been proposed including (a) peroxynitrite (ONOO^−^/ONOOH) formed by the reaction of oxygen‐ and nitrogen‐derived oxidants [superoxide anion, O^·−^, one electron reduced oxygen, and nitrogen monoxide (^·^NO)] and (b) other nitric‐oxide‐derived intermediates (reactive nitrogen species, RNS) generated by leukocyte peroxidase‐catalyzed biotransformation of nitrite in the presence of hydrogen peroxide [86, 93, 94, 95]. The contribution of a yet unidentified specific nitrase activity may also contribute to tyrosine nitration. The biological relevance of –NO_2_ protein‐incorporation is mainly attributed to the alteration of the physicochemical properties of tyrosine residue including phenol group pKa, redox potential, hydrophobicity, and volume (steric hindrance and distortion in protein conformation altering protein function) [96, 97]. Nitrated fibrinogen has been reported during acute and chronic inflammation and it has been suggested that it serves as oxidative risk biomarker in VTE linking inflammation and oxidant production to abnormal coagulation [90, 98, 99]. Vadseth *et al*., in 2004, and Parastatidis *et al*., in 2008, were able to show increased circulating levels of nitrated fibrinogen in patients with CAD and in otherwise healthy smokers respectively [90, 98]. They also reported that the impact of nitration was a biologically relevant gain‐of‐function in fibrinogen (opposed of the impact caused by oxidation) as evidenced by the increased fibrin aggregation kinetics, and a resulting clot with prothombotic phenotype presented with altered architecture and pore size, and changes in mechanical properties. Altered fibrin clot characteristics were also observed in clots formed with *in vitro* nitrated human fibrinogen when compared with clots formed with the non‐nitrated protein. Despite the fact that fibrinogen molecule contains a total of 39 tyrosine residues across the three fibrinogen chains, mass‐spectrometry‐based experiments by Medeiros *et al*. [100] indicated that less than nine nitrotyrosine residues were identified in clinical samples of ischemic stroke patients whereas Parastatidis *et al*. [98] revealed that *in vivo* tyrosine nitration in smokers is favored mainly at two residues, Tyr292 and tyr499, both located at the C‐terminal of β‐chain. It is also interesting to emphasize the finding that tyrosine nitration did not affect interaction with thrombin, neither with platelets or plasmin [98] and secondary structure of modified fibrinogen appeared to be unaffected by tyrosine nitration as observed by CD spectroscopy [98]. When nitrated fibrinogen molecules were removed by immunoaffinity the rate of fibrin polymerization and maximum absorbance were decreased compared to the fibrinogen that retained the nitrated fibrinogen molecules [98] providing a confirmation that nitrated fibrinogen was responsible for the functional fibrin alterations. Still unclear remains the mechanism(s) by which this chemical modification of only a small fraction of tyrosine residues causes this dramatic change in fibrinogen function. Speculation by the authors indicate that site‐specific tyrosine nitration results in conformational changes that enhance knob B:hole b interaction which was evidenced by the dose‐dependent association of tyrosine nitration levels and the accelerated knob B:hole b interactions when synthetic knob B mimetic peptides were used during polymerization. Alternatively, the modifications may have affected the stability of the molecule by structural alterations on the modified region introducing conformational changes upon binding of the knob B mimetics [98].

An interesting area for investigation is the potential overlap of fibrinogen tyrosine residues for nitration and phosphorylation. As discussed in a recently published review, Griswold‐Prenner *et al*. [101] unveiled 879 overlapping sites between nitration and phosphorylation in 460 proteins and suggested that these modifications may act in a complementary or competing mode and therefore interfering with downwards signaling pathways.

### Disulfide bonds modifications

Disulfide bond formation occurs when the sulfur atoms of two cysteine residues are joint by a covalent bond. Intramolecular disulfide bond formation is considered as one of the determinants of protein structure providing stability to a protein and is traditionally assumed to be completed during protein maturation. Moreover, it is considered that most of disulfide bonds are not a subject of reduction although in *in vitro* studies, Blomback *et al*. [102], showed the selective reduction of five disulfide bonds using the system thioredoxin/thioredoxin reductase. Crystal structure of mature human fibrinogen shows that the protein contains 29 disulphide bonds. In a recent study using differential cysteine alkylation followed by mass spectrometry analysis revealed that several structurally defined disulfide bridges were unpaired in mature circulating fibrinogen in the monomeric form but, they were turning to disulfides during the process of fibrin polymerization [103]. This dynamic process is of functional value since the alkylation of the unpaired cysteine residues led to altered clot structure potentially through the lower susceptibility of clot to the proteolytic mechanism whereas fibrin forming capacity was unaffected. It is interesting to note that these dynamically regulated bonds are located in the interior of the protein structure and therefore expected not to be easily accessible to oxidoreductases to manipulate their redox state. In the same study, the redox status of cysteine residues of fibrinogen secreted by hepatocytes in culture was also investigated and was found that fibrinogen was possessing the same disulfide status as the molecule *in vivo*. Based on these findings the authors concluded that the different disulfide‐bonded states of fibrinogen are predetermined and not a result of redox events after secretion.

### Lysine modifications

A variety of post‐translational modifications of lysine residues of fibrinogen molecule have been described and are considered critical for binding, activation and activity of fibrinolytic enzymes [104]. Lysine glycation involves a non‐enzymatic attachment of reducing sugars to lysine side chain and is present in patients with poorly controlled diabetes as well as of long duration [105, 106, 107]. Abnormal fibrin clot properties have been associated with diabetes [107]. Svensson *et al*. [108] found glycated three lysine residues at the position βK133, γK75, or γK85. Using proteomic analysis Bryk *et al*. [109] identified multiple sites of glycation in all three chains of fibrinogen in plasma fibrin clot of type 2 diabetes mellitus patients with four of the sites in Aα‐chain participating in factor XIIIa‐mediated cross‐linking and cleavage by plasmin. Site‐specific glycation of lysine residues could contribute to the altered structure/function of fibrin clots accounting at least in part for the reduced susceptibility of fibrinolysis most likely by decreased binding of fibrinolytic components as observed in diabetic patients [110, 111, 112]. In this way, glycation of fibrinogen may contribute to the increased risk for CVD in this patient population. Comprehensive reviews provide insights into the mechanisms of fibrinogen glycation, alterations in fibrin structure, and the contribution to thrombosis [78, 113].

Internal protein lysine acetylation is the transfer and addition of an acetyl group from acetyl‐coenzyme A to the side chain of a lysine residue by acetyltransferases. According to Svensson *et al*. [108], fibrinogen is acetylated at several lysine residues, in all three chains as identified by mass spectrometry in *in vitro* experiments some of which are involved in FXIIIa cross‐linking of fibrinogen and do not overlap with the glycated ones. Acetylation decreases the rate of fibrinogen polymerization but results in increased fiber diameters and susceptibility to fibrinolysis [110]. Aspirin (acetylsalicylic acid) can non‐enzymatically acetylate fibrinogen. In a purified fibrinogen model following the addition of aspirin or after treating patients with angina pectoris with acetylsalicylic acid the fibrin clots formed presented with altered clot architecture and became more prone to lysis [114, 115]. Therefore, acetylation of fibrinogen may provide insights into the mechanism of aspirin‐facilitated fibrin degradation and the potential to decrease the risk of thromboembolic events [116].

### Homocysteinylation

Homocysteinylation is the chemical incorporation of a sulfur‐containing product formed during methionine metabolism. Published studies propose that homocysteine acts as a harm of fibrin network altering fibrin clot structure and stability. The formation of abnormal clots could directly contribute to the increased risk of thrombosis in hyperhomocysteinemia [117, 118]. Homocysteinylation neutralizes the positive charge of lysine increasing its hydrophobicity with a potential impact on protein folding and solubility. In human fibrinogen, three lysine residues (AαLys562, BβLys344, γLys385) were shown to be homocysteinylated, *in vitro* and *in vivo* [119]. Homocysteinylation of fibrinogen negatively impacts protein function linking hyperhomocysteinemia with increased risk of thrombosis and atherosclerosis [118, 120, 121]. Sauls *et al*., showed that hyperhomocysteinemic rabbits form abnormal clots composed of thinner than normal fibers requiring a longer time for fibrin clots to lyse potentially due to impeded interaction of fibrinogen with fibrinolytic factors [122, 123]. The highly reactive cyclic ester of homocysteine, homocysteine thiolactone (HTL) reacts with the ε‐ΝΗ_2_ of lysine forming an isopeptide bond [124, 125, 126]. It has been shown that HTL‐treated fibrinogen displays a slower coagulation process, with tighter packed fibrin clots, characteristics that increase the likelihood of impaired fibrinolysis [127]. On the other hand, a study by Marchi *et al*. [128], in which fibrinogen isolated from plasma was used, demonstrated that very high concentrations of homocysteine are required in order to alter clot properties. Based on the above, it is apparent that the effect of homocysteinylation on fibrinogen biochemical properties requires further investigation.

### Glycosylation

Fibrinogen is a secreted protein undergoing site‐specific N‐glycosylation at Asn364 and Asn52 on Bβ‐ and γ‐chain respectively [129, 130]. Mechanistically, N‐glycans participate in inter‐protofibril lateral aggregation [8, 131]. The carbohydrate moieties are terminated by the covalent addition of one or two sialic acid units per carbohydrate. Increased content of sialic acid in the N‐glycans of the Bβ‐ and γ‐chains has been observed in patients with liver disease who also demonstrate impaired clotting capacity [6, 132, 133]. The addition of bulky molecules on Ββ‐ and γ‐chains results in clots with altered fibrin fibers [109, 134]. Brownlee *et al*. [135], reported that glycosylated fibrinogen is resistant to fibrinolysis most likely reflecting a steric impediment of plasmin binding to plasmin cleavage site on fibrinogen molecule.

### Citrullination

Citrullination is the enzymatic post‐translational deamination of arginine residues resulting in the formation of citrulline. Protein citrullination is associated with inflammatory diseases including rheumatoid arthritis (RA) and tumorigenesis. Citrulline represent an antigenic epitope recognized by RA‐specific autoantibodies and the citrullinated form of fibrinogen appears in RA synovial tissue [136]. Biochemically, the deamination of arginine results in loss of a positive charge with potential impact on hydrogen bond‐forming ability of the protein. The exact role of citrullination on fibrinogen function remains unknown however, based on published studies it can be speculated that citrullination may affect fibrinogen folding, polarity, fibrin clot formation, and may alter fibrin structure [137, 138]. Whether fibrinogen is citrullinated *in vivo* and what are the consequences of this modification are not established yet.

### Other fibrinogen PTMs

Cysteine residue S‐glutathionylation and S‐nitrosation have been reported only *in vitro*. Reduced, oxidized, and nitrosated glutathione resulted in altered fibrin polymerization with prolonged lag time, slower rates of protofibril aggregation and formation of clots with lower final turbidities relatively to unmodified fibrinogen most probably by inducing conformational changes through interaction with specific domain in the αC region of fibrinogen [139, 140]. In a meta‐analysis study, Sovova *et al*. [141] used molecular dynamic simulation aiming to characterize the impact of several PTMs, reported in the literature, on the behavior of fibrinogen coiled coil domain. Based on the effect of citrullination and methionine oxidation the authors proposed that certain PTMs alter fibrinogen secondary structure.

## Future perspectives

Fibrin clot formation and lysis are finely tuned dynamic processes which secure that no bleeding or thrombotic events will occur. Fibrinogen is a central component of the hemostatic process, thus, the mechanisms that control the biological function of this protein in health and disease represent hot areas of investigation.

**Fig. 5:**
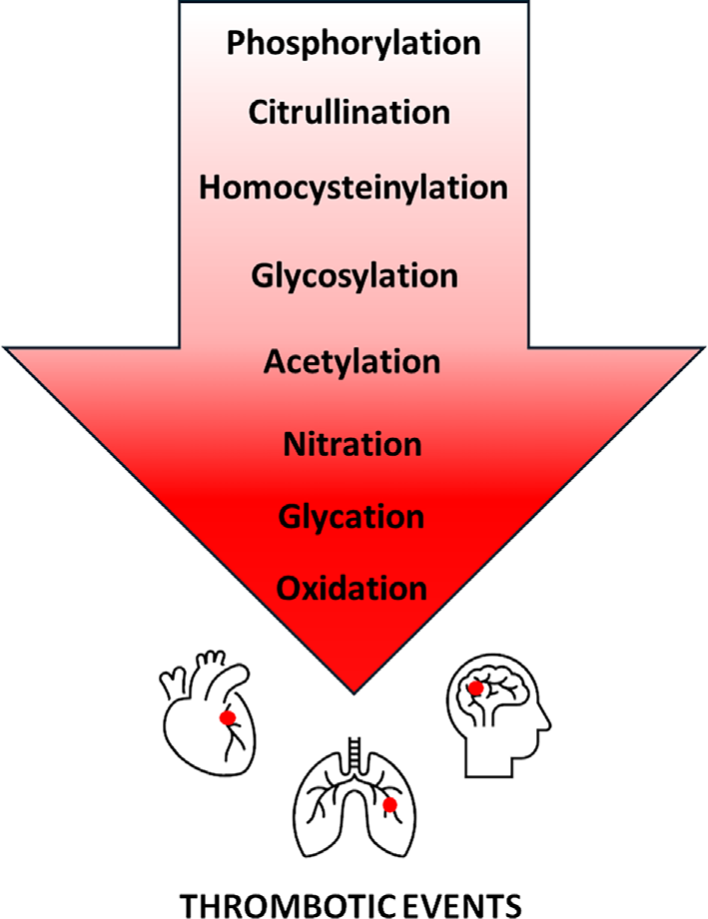
List of fibrinogen modifications with clinical evidence. Light to dark red color gradient represents fibrinogen post‐translational modifications with clinical evidence of their impact on thrombotic events. Red circle: Abnormal thrombus/thromboembolus.

**Fig. 6:**
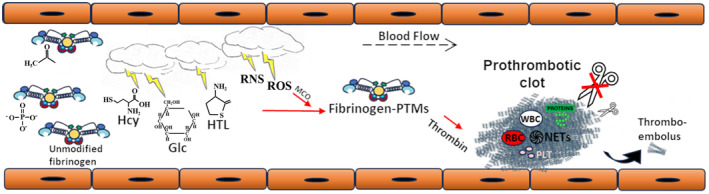
Proposed mechanism of *in vivo* intravascular clot formation under the influence of PTMs. Fibrinogen PTMs arising before secretion or within the circulation lead to the formation of a thrombus with a potentially prothrombotic phenotype, including altered fibrin clot network architecture, reduced susceptibility to lysis and altered viscoelastic properties. Clinical consequences such as venous thromboembolism may occur. −COCH₃, acetyl group; Glc, glucose; Hcy, homocysteine; HTL, homocysteine thiolactone; NETs, neutrophil extracellular traps; PLT, platelets; ${\mathrm{PO}}_{4}^{‐3}$, phosphate group; RBC, red blood cells; RNS, reactive nitrogen species; ROS, reactive oxygen species; WBC, white blood cells.

Post‐translational modifications provide spatial and temporal control of protein function. A wealth of knowledge regarding PTMs that target fibrinogen is currently available while most of these data were derived through mass‐spectrometry‐based proteomic studies. Figure 5 presents experimentally detected fibrinogen PTMs for which there is clinical evidence regarding their impact on thrombotic events and are hierarchically placed from “lighter to darker red area” relatively to existing evidence. Specific PTMs that are not placed in red area may alternatively participate in crucial signaling events in the milieu of the thrombus. Fibrinogen is a relatively abundant protein with a half‐life in circulation of about 4 days. Thus, it is logical to assume that PTMs represent additional regulatory mechanisms that control intravascular fibrinogen clotting and its mechanical properties, and impact biological stability and fibrinogen ability to interact with proteins, cells and other plasma components (Fig. 6). The potential interplay between different modifications has not been investigated yet. It has been recently documented that site‐specific nitration and phosphorylation may occur on the same tyrosine residues on several proteins [101]. A reasonable hypothesis is that fibrinogen displays different PTMs in health and disease. Thus, reciprocal targeting by different modifications may represent a “switch” that turns the molecule from physiological to prothrombotic.

Another interesting concept is the impact of PTMs on fibrinogen interactions with other blood components and the endothelium. Besides its polymerization, fibrinogen acts as a scaffold protein during clot formation. Protein‐to‐protein interactions are typically based on electrostatic interactions occurring on the surface of the proteins. In this regard, PTMs will introduce extra charge or will neutralize existing charge on protein surface. Thus, post‐translationally modified fibrinogen will display altered capacity to interact with proteins. The pathophysiological consequences of these phenomena are yet unknown. The comprehensive analysis of thrombus composition and the qualitative and quantitative changes in thrombus components induced by post‐translationally modified fibrinogen represent the first step to link post‐translationally modified fibrinogen and thrombotic phenotype.

## Conflict of interest

The author declares no conflict of interest.
